# Psoralen Induces Developmental Toxicity in Zebrafish Embryos/Larvae Through Oxidative Stress, Apoptosis, and Energy Metabolism Disorder

**DOI:** 10.3389/fphar.2018.01457

**Published:** 2018-12-18

**Authors:** Qing Xia, Lingying Wei, Yun Zhang, Haotian Kong, Yongping Shi, Xue Wang, Xiqiang Chen, Liwen Han, Kechun Liu

**Affiliations:** ^1^Biology Institute, Qilu University of Technology (Shandong Academy of Sciences), Jinan, China; ^2^School of Pharmacy, Shanxi Medical University, Taiyuan, China

**Keywords:** psoralen, zebrafish, developmental toxicity, oxidative stress, apoptosis, energy metabolism disorder

## Abstract

Psoralen toxicity is an issue of wide concern. However, an assay for psoralen-induced developmental toxicity has not been reported to date. Moreover, the underlying mechanism of psoralen-induced developmental toxicity is unclear. Therefore, this study attempted to develop a psoralen-induced developmental toxicity assay in zebrafish embryos/larvae. Psoralen treatment caused a decrease in the hatching rate and body length and a significant increase in the malformation rate of zebrafish. Yolk retention, pericardial edema, swim-bladder deficiency, and curved body shape were also observed after psoralen treatment. Yolk retention might have been caused by an abnormality in lipid metabolism. Further experiments indicated that psoralen exerted toxic effects on the developing heart, liver, phagocytes, and nervous system. Increased generation of reactive oxygen species, inhibition of total superoxide dismutase activity, and increased malondialdehyde concentrations indicated inhibition of antioxidant capacity and the presence of oxidative stress. A greater number of apoptotic cells were observed after psoralen exposure, relative to the control. Furthermore, the results of gene-expression analysis showed that psoralen induced developmental toxicity by means of oxidative stress, apoptosis, and energy metabolism abnormalities. These findings will be helpful in understanding psoralen-induced toxicity.

## Introduction

Psoralen an active compound of Chinese herbs such as *Psoralea corylifolia* L., which is widely used in traditional medicine for treatment of psoriasis, vitiligo, osteoporosis, osteosarcoma, bone fracture, and osteomalacia (Liu et al., 2004; Adişen et al., 2010; Lu et al., 2014; Doppalapudi et al., 2017). Previous studies have demonstrated that psoralen possesses extensive pharmacological properties, including anti-cancer (Yi et al., 2011; Wu et al., 2013; Wang et al., 2016), anti-osteoporosis (Yuan et al., 2016; Chen et al., 2017; Li F. et al., 2017; Zheng et al., 2017), anti-inflammatory (Li et al., 2018; Xiao-Tian et al., 2018), anti-fungal (Yu et al., 2017), anti-depressant (Xu et al., 2008), and antibacterial (Shim et al., 2009) properties. Psoralen polymer-lipid hybrid nanoparticles cause reversal of multidrug resistance in MCF-7/ADR cells (Huang Q. et al., 2018). The toxicity of psoralen has recently come under the microscope, and hepatotoxicity of psoralen has recently been observed *in vitro* and *in vivo* (Diawara et al., 2000; Hai et al., 2017; Li Z.J. et al., 2017). As recorded in *Bencao Haili* and *Deipei Bencao*, two Chinese medical classic books, pregnant women should be caution with psoralen. However, there are currently no reports on the developmental toxicity of psoralen.

Pleiotropic deleterious effects of oxidative stress are implicated in a variety of chemical-induced toxicities. The presence of high levels of reactive oxygen species (ROS) can destroy the defense system of an organism, with consequences including damage of critical cellular components such as DNA, lipid, and protein macromolecules. Subsequent mitochondrial dysfunction and impairment of energy production can induce cytochrome c release, p53 accumulation, caspase activation, and, ultimately, cell death (Deavall et al., 2012; Pereira et al., 2012; Kupsco and Schlenk, 2015). Cellular energy is also crucial for cell growth, division, and differentiation in the course of early embryo development (Don, 1992; Gardner et al., 2000; van Dartel et al., 2014).

Numerous advantages of zebrafish, such as their high fecundity, short generation time, and transparent body, make them an accredited model for conducting developmental toxicity assays. Zebrafish share genetic, physiological, and anatomical homology with humans. Furthermore, chemical-induced malformations in zebrafish can be observed directly under a stereomicroscope (Sarvaiya et al., 2014; Nishimura et al., 2016; Huang M. et al., 2018; Qian et al., 2018).

In the present study, zebrafish embryos/larvae at 4 h post-fertilization (hpf) were used to assess psoralen-induced developmental toxicity until 96 hpf. The antioxidant capacity, oxidative stress status, and apoptosis levels of the larvae were analyzed. Moreover, the effects of psoralen on expression of genes related to oxidative stress, apoptosis, and energy metabolism were investigated. This study provides a better understanding of psoralen-induced developmental toxicity and the underlying molecular mechanisms.

## Materials and Methods

### Chemicals

Psoralen was purchased from the National Institutes for Food and Drug Control (110739-201617) (Beijing, China). Stock solutions were prepared in dimethyl sulfoxide, and serial dilutions were prepared in embryo water (5 mM NaCl, 0.17 mM KCl, 0.4 mM CaCl_2_, and 0.16 mM MgSO_4_) before the experiments. All other chemicals and reagents used in this study were of analytical grade.

### Zebrafish Husbandry and Embryo Collection

The zebrafish were obtained from Zebrafish Drug Screening Platform of Shandong Academy of Sciences. The adult zebrafish AB strain and the *Tg(cmlc2:EGFP), Tg(L-FABP:EGFP), Tg(Lyz:EGFP), Tg(Vmat:GFP)* transgenic zebrafish lines used in this study were maintained at 28 ± 0.5°C with a 14:10-h light-dark cycle in an automatic zebrafish housing system (ESEN, Beijing, China). The fish were fed live brine shrimp twice a day. Two male and one female zebrafish were separated in a spawning box on the night before spawning. In the morning, fertilized eggs were collected, washed three times with fish water, and then maintained in a light incubator at 28°C until 4 hpf.

### Lethal and Teratogenic Assay

Normal developing embryos were selected under a stereomicroscope (SZX16, Olympus, Tokyo, Japan) at 4 hpf and randomly placed into 24-well plates at a density of 15 per well. Serial concentrations of psoralen were added into each well to a final volume of 2 mL. The exposure solutions were replaced every 24 h until 96 hpf. Meanwhile, dead embryos were removed, and the mortality in each well was recorded. Thereafter, lethal curves at 24, 48, 72, and 96 hpf were established, and the values of 10% lethal concentration (LC_10_) and 1% lethal concentration (LC_1_) were calculated. In subsequent experiments, psoralen was used at concentrations of 1/10 LC_1_, 1/3 LC_1_, LC_1_, and LC_10_.

As described above, normal developing embryos were exposed to psoralen at concentrations of 1/10 LC_1_, 1/3 LC_1_, LC_1_, and LC_10_. These embryos were observed for phenotypic changes and photographed at 24, 48, 72, and 96 hpf. Hatching rates at 48 and 72 hpf were recorded, and the malformation rate at 96 hpf was calculated. Finally, all larvae were photographed for assessment of body length.

### Assessment of the Effect of Psoralen on the Developing Heart

The effect of psoralen on the morphology and function of the developing heart was assessed in the *Tg(cmlc2:EGFP)* expression line (Huang et al., 2003). The morphology and cardiac function of the zebrafish heart were almost fully developed at 72 hpf. Therefore, the heart rate, area of pericardial edema, and distance between the cardiac sinus venosus and bulbus arteriosus (SV–BA) were measured at 72 hpf. We recorded the heart beating for 20 s under a microscope. The number of heart beats in a 20 s period was multiplied by 3 to calculate heart rate. Each larva was pictured under a fluorescence stereomicroscope (AXIO Zoom.V16, ZEISS, Oberkochen, Germany). Area of pericardial edema and SV-BA distance were measured using Image-Pro Plus software (Media Cybernetics, Bethesda, United States).

### Assessment of the Effect of Psoralen on the Developing Liver

Zebrafish of the *Tg(L-FABP:EGFP)* transgenic line express the enhanced green fluorescent protein (EGFP) in the liver (Her et al., 2003). At 96 hpf, lateral photographs of each larva in the control and treatment groups were acquired using a fluorescence stereomicroscope. Then, the area and fluorescence intensity of the liver were determined using the Image-Pro Plus software.

### Assessment of the Effect of Psoralen on Phagocytes

Phagocytes are an important component of the immune system, and phagocyte number is closely related to immune responses, inflammation, and foreign-body responses. Zebrafish of the *Tg(Lyz:EGFP)* transgenic line express EGFP in macrophages and neutrophils, which makes it easy to quantitate the macrophage and neutrophil number in zebrafish larvae (Hall et al., 2007). In this experiment, control and psoralen-treated transgenic zebrafish were photographed until 72 hpf, and the total number of macrophages and neutrophils was counted manually.

### Assessment of the Effect of Psoralen on the Developing Nervous System

The vesicular monoamine transporter of the *Tg(Vmat:GFP)* line of zebrafish was labeled by the green fluorescent protein during embryonic development (Wu et al., 2016). At 96 hpf, photographs of larvae in the control and treatment groups were taken from the top view, and the length of dopamine ganglia was measured using the Image-Pro Plus software. Furthermore, the total swimming distance, swimming velocity and motion track of each control and psoralen-treated larva was analyzed until 7 days post-fertilization using Zebralab (Viewpoint, Lyon, France).

### Measurement of Reactive Oxygen Species Generation

Generation of reactive oxygen species (ROS) in control and psoralen-treated zebrafish was detected at 96 hpf using an ROS assay kit (Nanjing Jiancheng Bioengineering Institute, Nanjing, China) based on detection of the fluorescent probe dichloro-dihydro-fluorescein diacetate (DCFH-DA). After psoralen treatment, the larvae were incubated with 30 μM DCFH-DA for 40 min in the dark at 28 ± 0.5°C. They were then washed with phosphate-buffered saline solution (PBS) three times and anesthetized with 0.16% tricaine. A lateral image of each larva was acquired using a fluorescence microscope, and the fluorescence intensity was quantified using the Image-Pro Plus software.

### Detection of Apoptotic Cells

Apoptotic cells from larvae in each group were detected by staining with acridine orange. In brief, after psoralen treatment (at 96 hpf), the larvae were incubated with 10 μg/mL acridine orange staining solution in the dark for 30 min. They were then washed with PBS three times and anesthetized with 0.16% tricaine. A lateral image of each larva was acquired within 20 min using a fluorescence microscope.

### Assessment of Antioxidative Enzyme and Lipid Peroxidation Activities

In this study, 50 larvae in each group were pooled together in cold saline and homogenized on ice at 96 hpf. The supernatants were collected for analysis of antioxidative enzyme and lipid peroxidation activities after centrifugation at 3,500 rpm for 15 min at 4°C. Total superoxide dismutase (T-SOD) activity and malondialdehyde (MDA) levels were assessed using commercial kits (Nanjing Jiancheng Biotechnology Institute, China) in accordance with the manufacturer’s protocols.

### Real-Time Quantitative PCR Assay

After psoralen-treatment (at 96 hpf), total RNA was extracted from 30 larvae using the TRIzol reagent (Invitrogen, Waltham, United Status). The quality of extracted RNA was evaluated on the basis of OD_260_/OD_280_ ratio. Then, cDNA was synthesized using the HiScript II Q RT SuperMix (Vazyme, Nanjing, China), and quantitative real-time polymerase chain reaction (RT-PCR) was performed with an RT-PCR system (Biorad, CA, United Status) using the SYBR Green mix (Takara, Dalian, China). The primer sequences of the genes that were detected are shown in Table 1.

**Table 1:** The sequences of primer pairs used in real-time quantitative PCR assay.

No.	Gene symbol	Forward primer	Reverse primer
1	*keap1*	ACATGGAGTCTCAG TCTACC	GGCATATCTGTTACA AGCGT
2	*nrf2*	CACCCAACATGAATC AACTG	ATTTCCGCCATCTGA TGTAAT
3	*Mn-Sod*	TAGATGTCTGGGA ACATGCG	TGGCTTTAACATAG TCCGGTC
4	*Cu/Zn-Sod*	GGTGGCAAT GAGGAAAGTC	ATCACTCCACA GGCCAGA
5	*p53*	AGAATCGTGAATCATC TGAGC	CATCACCTTAATCAGA GTCGC
6	*puma*	GCTGGAAGTTACATG ATACCG	CGCAGTTATTGCTCCT GTAAG
7	*bax*	GGAAGAGAAAGAGTTG GAGACA	GGCTTGAACCATCTA CATCTG
8	*bcl-2*	TGCACACTGGATGAC TGACTA	TGACCGTACATCTC CACGAA
9	*apaf-1*	GCTGGGTGACTGTAT TTGG	ACACTCCTTAATGAGT GAACG
10	*caspase-9*	CTGGAAACTCTTGT CAGAATGG	TTCAATGCCTTGAC GAGGTTTA
11	*caspase-3*	ATGACCAGGGTCAA CCATAA	AAGTACATCTCTT TGGTGAGC
12	*hmgcra*	GGCAAGTCGCAACT TGTAT	GTTCTGTGCCCTTT GAGAT
13	*pparα1*	CAAGTGCCAATA CTGTCGAT	TCTCTGCCTTCAAC CTTAGC
14	*fas*	GAAAGTACTGTCCATT CCCAGG	GAGGGGAGCGCAT GATTTCT
15	*β-actin*	CTCCGGTATGTG CAAAGC	CCATCACTCCCTG ATGTCT


### Statistical Analysis

The results were expressed as mean ± standard deviation and processed by one-way analysis of variance. Comparison between the groups was performed using Student–Newman–Keuls (SNK) method. Differences from controls were considered significant when *p* was less than 0.05 or 0.01.

## Results

### Lethal and Teratogenic Effects of Psoralen

**FIGURE 1:**
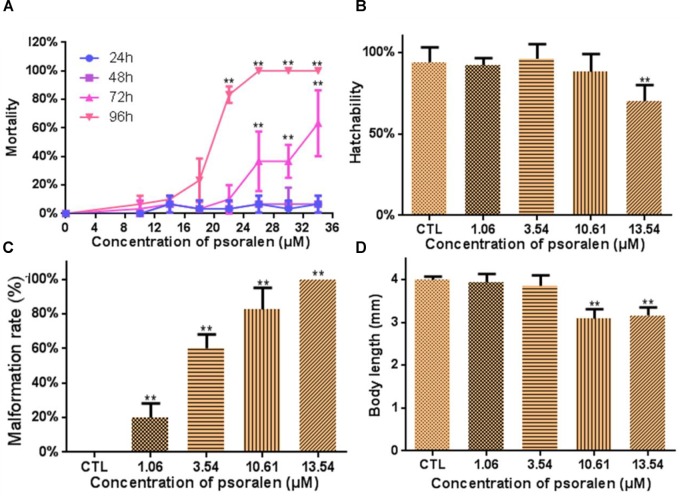
Developmental toxicity of psoralen in zebrafish larvae. **(A)** Mortality curves until 24, 48, 72, and 96 hpf. **(B)** Hatching rates at 72 hpf. **(C)** Malformation rates at 96 hpf. **(D)** Body length at 96 hpf. The values are expressed as mean ± SD (*n* = 3). ^∗^Represents *p*-value less than 0.05 and ^∗∗^represents *p*-value less than 0.01.

Figure 1A shows the lethal effects of psoralen at 24, 48, 72, and 96 hpf. Mortality rates in the treatment group exhibited a dose- and time-dependent increase. The values of LC_50_, LC_10_, and LC_1_ at 96 hpf were determined to be 18.24, 13.54, and 10.61 μM. The hatching rate in the 13.54-μM psoralen group (70%) was much lower than that in the control group (94%; Figure 1B). Malformation rates exhibited a significant increase in a dose-dependent manner (Figure 1C). All of the larvae in the 13.54-μM psoralen group exhibited teratogenic effects. The body length of larvae in the 10.61- and 13.54-μM psoralen-treatment groups was notably shorter than that in the control group (Figure 1D).

**FIGURE 2:**
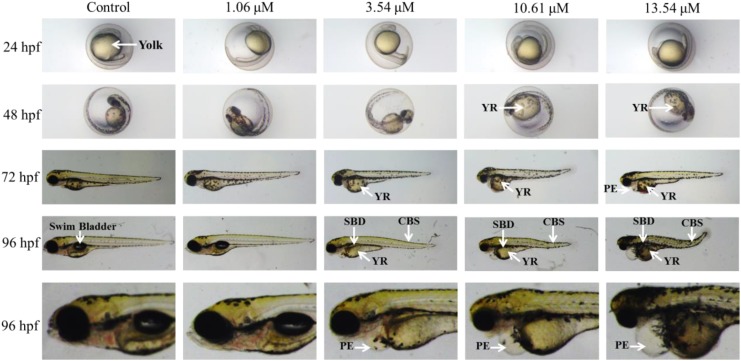
The phenotypic changes of embryos at 24, 48, 72, and 96 hpf. SBD, swim bladder deficiency; YR, yolk retention; PE, pericardial edema; CBS, curved body shape.

A variety of morphological abnormalities were observed in the psoralen-treatment groups from 24 to 96 hpf, including yolk retention, swim-bladder deficiency, pericardial edema, and curved body shape (Figure 2). Yolk retention and pericardial edema were the most pronounced morphological alterations. Yolk retention was first observed in the 10.61- and 13.54-μM psoralen-treatment groups at 48 hpf; at 72 and 96 hpf, yolk retention was also observed in the 3.54-μM psoralen-treatment group. Pericardial edema was first observed in the 13.54-μM psoralen-treatment group at 72 hpf. At 96 hpf, pericardial edema was found to be prevalent in the 3.54, 10.61, and 13.54 μM psoralen-treatment groups.

### Effects of Psoralen on the Developing Heart

**FIGURE 3:**
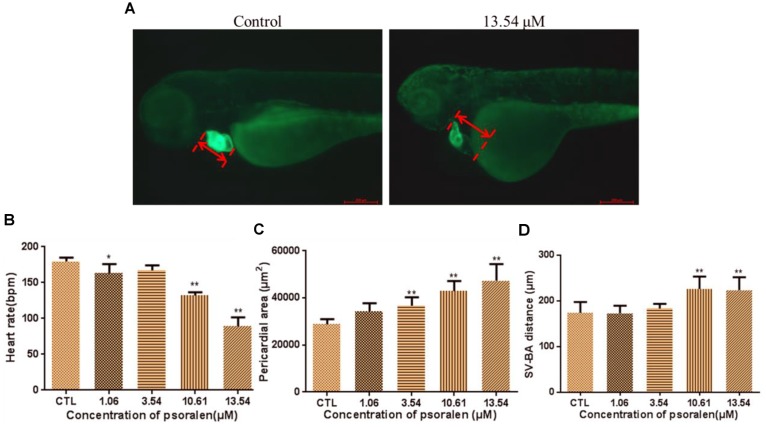
Effects of psoralen on developing heart. **(A)** Phenotypes of larvae of *Tg(cmlc2:EGFP)* lines. SV–BA distance is indicated by red row. **(B)** Heart rates at 72 hpf. **(C)** Pericardial area at 72 hpf. **(D)** SV–BA distance at 72 hpf. The values are expressed as mean ± SD (*n* = 3). ^∗^Represents *p*-value less than 0.05 and ^∗∗^represents *p*-value less than 0.01.

The phenotypes of larvae of the *Tg(cmlc2:EGFP)* transgenic line are shown in Figure 3A. The heart rates of larvae in the psoralen-treatment groups were significantly decreased relative to those in the control group (Figure 3B). In the 13.54-μM psoralen-treatment group, especially, the heart rate had reduced to 89 ± 12 bpm, which was significantly lower compared to that in the control group (180 ± 5 bpm). The pericardial area had increased in extent in a dose-dependent manner after psoralen treatment (Figure 3C). The SV–BA distances in the 10.61 and 13.54 μM psoralen-treatment groups had increased significantly relative to that the control group (Figure 3D).

### Effects of Psoralen on the Developing Liver

**FIGURE 4:**
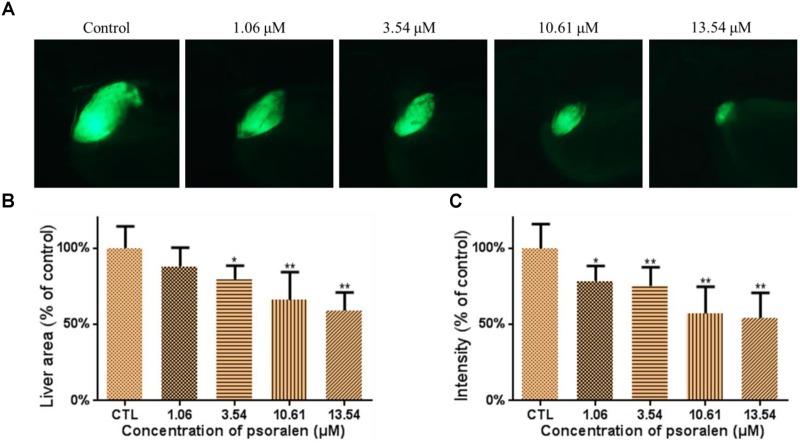
Effects of psoralen on developing liver. **(A)** Phenotypes of larvae of *Tg(L-FABP:EGFP)* lines. **(B)** Liver area at 96 hpf. **(C)** Liver intensity at 96 hpf. The values are expressed as mean ± SD (*n* = 3). ^∗^Represents *p*-value less than 0.05 and ^∗∗^represents *p*-value less than 0.01.

The effects of psoralen on the developing liver were assessed using the larvae of the *Tg(L-FABP:EGFP)* transgenic line. As shown in Figure 4A, psoralen was toxic to the developing liver. The liver area and fluorescence intensity had both decreased in a dose-dependent manner after psoralen treatment. The liver area in the 13.54-μM psoralen-treatment group had decreased to 59.16 ± 11.78% of that of the control group (Figure 4B). As shown in Figure 4C, the liver fluorescence intensity in the 13.54 μM psoralen-treatment group had decreased to 54.27 ± 16.37% of that of the control group.

### Effects of Psoralen on Total Number of Macrophage and Neutrophil

**FIGURE 5:**
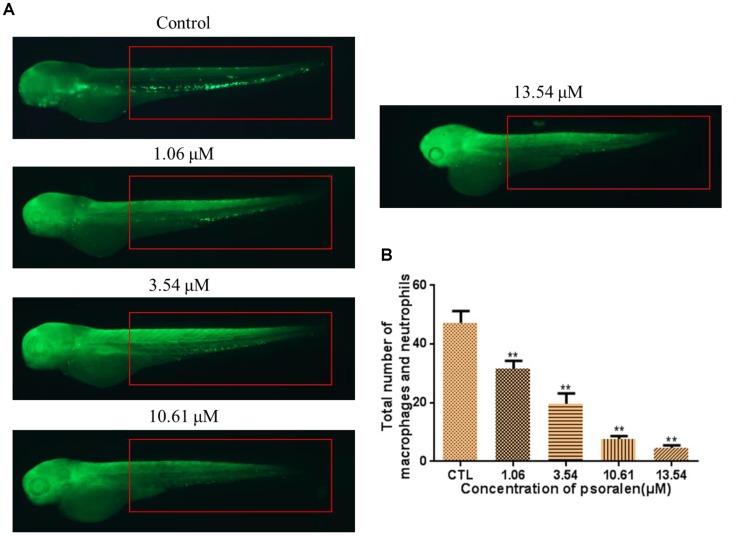
Effects of psoralen on the macrophage and neutrophil. **(A)** Phenotypes of larvae of *Tg(Lyz:EGFP)* lines. The total number of macrophage and neutrophil was counted in red box regions. **(B)** Total number of macrophage and neutrophil in larva at 72 hpf. The values are expressed as mean ± SD (*n* = 3). ^∗^Represents *p*-value less than 0.05 and ^∗∗^represents *p*-value less than 0.01.

As shown in Figure 5, the total number of macrophages and neutrophils in the larvae had decreased significantly in a dose-dependent manner after psoralen treatment. At 96 hpf, the total number of macrophages and neutrophils in the control group had reached 47 ± 4; in contrast, in the 1.06 μM psoralen-treatment group, the total number of macrophages and neutrophils had only reached 32 ± 3. Only 5 ± 1 macrophages and neutrophils were detected when the concentration of psoralen was increased to 13.54 μM.

### Effects of Psoralen on the Developing Nervous System

**FIGURE 6:**
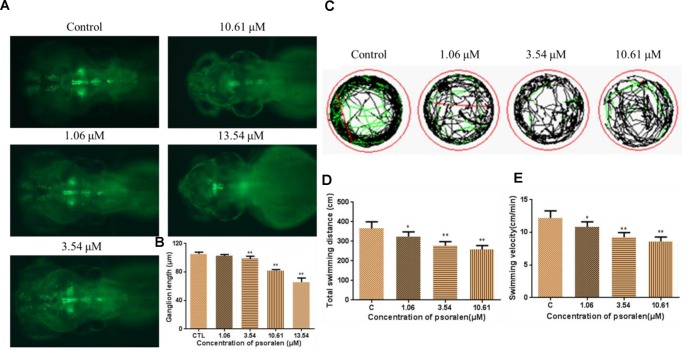
Effects of psoralen on the developing nervous system. **(A)** Dopamine ganglion at 96 hpf. **(B)** The length of dopamine ganglion. **(C)** Motion track of larva at 7 dpf, red lines indicate high speed, green lines indicate middle speed, black lines indicate slow speed. **(D)** Total swimming distance. **(E)** Swimming velocity. The values are expressed as mean ± SD (*n* = 3). ^∗^Represents *p*-value less than 0.05 and ^∗∗^represents *p*-value less than 0.01.

As shown in Figure 6A, the psoralen-treatment groups exhibited an obvious decrease in the fluorescence intensity of Vmat–GFP relative to the control group. They also exhibited a significant decrease in the length of dopamine ganglia relative to the control group (Figure 6B). Furthermore, the total swimming distance and velocity in the treatment groups had significantly decreased in a dose-dependent manner (Figures 6C–E).

### Effects of Psoralen on ROS Generation

**FIGURE 7:**
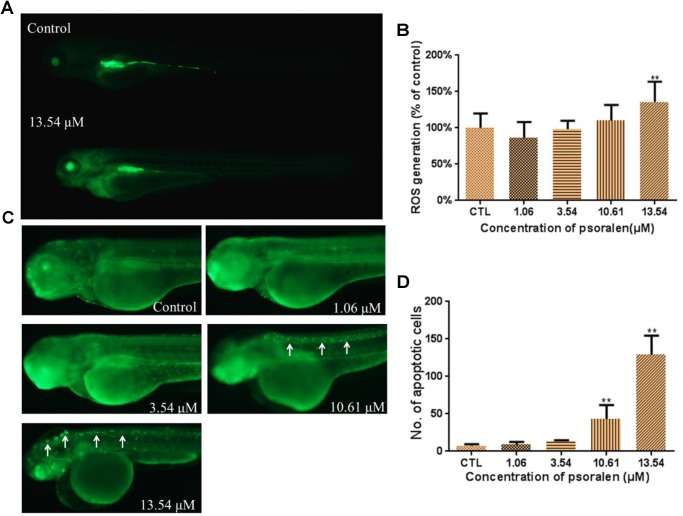
Effects of psoralen on the ROS generation and apoptosis. **(A)** Fluorescence micrographs of ROS generation in zebrafish larvae at 96 hpf. **(B)** Quantitative analysis of ROS generation. **(C)** Fluorescence micrographs of apoptotic cells in zebrafish larvae at 96 hpf (White arrows indicate apoptotic cells in nervous system). **(D)** Quantitative analysis of apoptotic cells. The values are expressed as mean ± SD (*n* = 3). ^∗^Represents *p*-value less than 0.05 and ^∗∗^ represents *p*-value less than 0.01.

As shown in Figure 7A, the larvae in the treatment groups exhibited markedly higher fluorescence intensities than those in the control group, which suggested that ROS were generated after psoralen exposure. A significant increase in ROS generation was observed in the 13.54 μM psoralen-treatment group (Figure 7B).

### Effects of Psoralen on Apoptosis

The number of apoptotic cells in the larvae of the 10.61 and 13.54 μM psoralen-treatment groups were significantly increased relative to that in the control larvae (Figures 7C,D). In the 10.61 μM psoralen-treatment group, the apoptotic cells were mainly distributed in the neurocoel. In the 13.54 μM psoralen treatment group, the apoptotic cells were distributed almost throughout the entire body, especially in the neurocoel and brain areas.

### Effects of Psoralen on T-SOD Activity and MDA Levels

**FIGURE 8:**
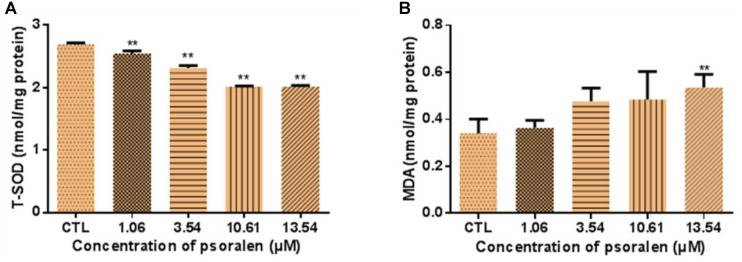
Effects of psoralen on activity of T-SOD and MDA Content in zebrafish larvae at 96 hpf. **(A)** T-SOD activities. **(B)** MDA content. The values are expressed as mean ± SD (*n* = 3, which means 3 pools of 50 larvae). ^∗^Represents *p*-value less than 0.05 and ^∗∗^represents *p*-value less than 0.01.

As shown in Figure 8A, larvae treated with 1.06, 3.54, 10.61, and 13.54 μM psoralen exhibited a significant decrease in T-SOD activity in a dose-dependent manner, which indicated that the antioxidant capacity of the larvae had decreased. Moreover, the MDA levels of larvae in the 13.54 μM psoralen treatment group were significantly increased relative to those in the control group, which indicated that lipid peroxidation had occurred (Figure 8B).

### Effects of Psoralen on Gene Expression

**FIGURE 9:**
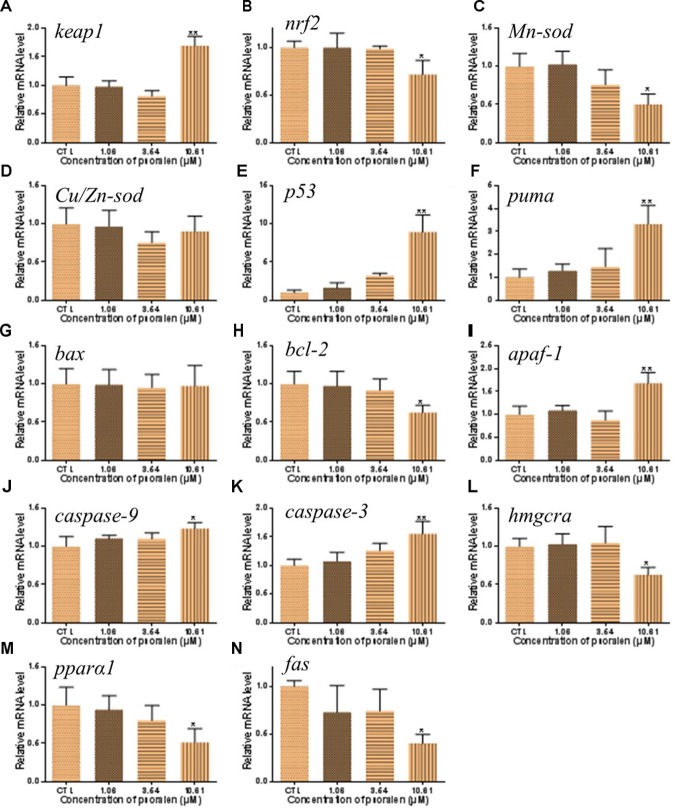
Effects of psoralen on gene expression in zebrafish larvae at 96 hpf. The mRNA levels of *keap1* **(A)**, *nrf2* **(B)**, *Mn-sod* **(C)**, *Cu/Zn-sod* **(D)**, *p53* **(E)**, *puma* **(F)**, *bax* **(G)**, *bcl-2* **(H)**, *apaf-1* **(I)**, *caspase-9* **(J)**, *caspase-3* **(K)**, *hmgcra* **(L)**, *pparα1* **(M)**, *fas* **(N)**. The values are expressed as mean ± SD (*n* = 3, which means 3 pools of 30 larvae). ^∗^Represents *p*-value less than 0.05 and ^∗∗^represents *p*-value less than 0.01.

In order to investigate the mechanisms of psoralen-induced developmental toxicity, the mRNA expression levels of genes related to antioxidant activities, apoptosis, and energy metabolism were measured. In the 10.61 μM psoralen treatment group, the expression level of the gene encoding the Kelch-like ECH-associated protein 1 (*Keap1*) was significantly increased relative to that in the control (Figure 9A). In the psoralen treatment groups, the expression levels of genes encoding nuclear factor erythroid-derived 2-like 2 (*Nrf2*; Figure 9B) and manganese superoxide dismutase (*Mn-Sod*; Figure 9C) were decreased relative to the control; however, no obvious change was detected in the expression level of the gene encoding copper/zinc superoxide dismutase (*Cu/Zn-Sod*; Figure 9D). Psoralen treatment caused no significant change in the expression level of the gene encoding Bcl-2 associated X protein (*Bax*) (Figure 9G). In the 10.61 μM psoralen-treatment group, the expression levels of genes encoding p53 protein (*p53*; Figure 9E), p53 upregulated modulator of apoptosis (*puma*; Figure 9F), B-cell lymphoma-2 (*bcl-2*; Figure 9H), apoptotic protease activating factor 1 (*apaf-1*; Figure 9I), cysteinyl aspartate specific proteinase 9 (*caspase-9*; Figure 9J), and cysteinyl aspartate specific proteinase 3 (*caspase-3*; Figure 9K) were significantly increased relative to the control. In contrast, the expression levels of genes encoding hydroxymethyl glutaryl coenzyme A reductase a (*hmgcra*; Figure 9L), peroxisome proliferator-activated receptor alpha 1 (*pparα1*; Figure 9M), and fatty acid synthase (*fas*; Figure 9N) were downregulated in the 10.61-μM psoralen-treatment group.

## Discussion

Psoralen is a well-known active compound present in psoraleae fructus (Bu Gu Zhi), a traditional Chinese medicine comprising the dried ripe fruit of *Psoralea corylifolia L.* (Lu et al., 2014; Zheng et al., 2017). Previous studies have demonstrated that psoralen induces hepatotoxicity in mice (Hai et al., 2017). In the present study, we assessed the developmental toxicity of psoralen in zebrafish embryos/larvae. The zebrafish is an ideal alternative model for assessment of developmental toxicity. The development process of zebrafish is highly conserved across vertebrates, and it is easy to record this development process *in vitro* (Nishimura et al., 2016). In our study, psoralen treatment induced a dose- and time-dependent increase in the mortality of zebrafish embryos/larvae from 4 to 96 hpf. Psoralen also inhibited hatching and growth (in terms of body length) and caused an apparent and dose-dependent increase in the malformation rate of zebrafish embryos/larvae. Upon psoralen treatment, yolk retention was observed first, followed by pericardial edema, swim-bladder deficiency, and curved body shape. These results indicated that psoralen exposure induced developmental toxicity in these zebrafish. Further, we investigated the toxic effects of psoralen on the developing heart, liver, phagocytes, and nervous system. The reduced heart rate in the psoralen-treatment groups denoted cardiac dysfunction, while the increased pericardial area and SV–BA distance indicated psoralen-induced structural abnormalities of the heart. The decreased liver area and fluorescence intensity in the psoralen-treatment groups indicated hepatocyte damage. Macrophages play a key role in host defense in innate and adaptive immunity (Varin and Gordon, 2009). Neutrophils inform and shape immune responses, contribute to the repair of tissue as well as its breakdown (Nathan, 2006). The remarkable reduction of the total number of macrophages and neutrophils in psoralen-treated zebrafish in the present study indicated that their immune system was severely compromised. The inhibition of dopamine ganglia and swimming ability in psoralen-treated zebrafish indicated the toxic effect of psoralen on the developing nervous system.

Oxidative stress is regarded as a mechanism of chemical-induced toxicity. Cell survival is maintained through the balance of ROS levels and cellular antioxidant capacity (Lee et al., 2017). In the present study, the psoralen-treatment groups exhibited an increased generation of ROS; however, the activity of T-SOD in these groups was reduced, and the MDA levels were significantly increased. These results indicated that psoralen treatment induced oxidative stress in the zebrafish embryos/larvae during development. Because oxidative stress can result in apoptosis, we investigated the presence of apoptotic cells by acridine orange staining (Corcoran et al., 1994; Kupsco and Schlenk, 2015). Despite the fact that apoptosis can occur naturally during embryogenesis, we observed an obvious increase in apoptotic cells in psoralen-treated larvae. These apoptotic cells were densely distributed in the neurocoel and brain area. These results demonstrated that nerve cells might be highly sensitive to psoralen.

Oxidative stress, apoptosis, and energy metabolism abnormalities during embryogenesis can result in malformation (van Dartel et al., 2014; Kupsco and Schlenk, 2015). The yolk is the only resource of energy for zebrafish embryos/larvae during the period of development. In this study, yolk retention was first observed at 48 hpf. This retention might have been caused by abnormalities in energy metabolism. Energy deficiency might induce organ developmental abnormality. The roles of energy deficiency on psoralen-induced organ developmental toxicity should be discussed in future assay. In order to investigate the underlying mechanism of psoralen-induced developmental toxicity, we determined the expression levels of genes related to oxidative stress, apoptosis, and energy metabolism. The *Nrf2*–*Keap1* system acts as a defender against oxidative stress (Suzuki and Yamamoto, 2017; Bellezza et al., 2018). *Keap1*, an endogenous inhibitor of *Nrf2*, regulates the activity of *Nrf2* and acts as a sensor for oxidative stresses (Toyama et al., 2007; Copple et al., 2008). In the present study, psoralen-treated zebrafish larvae exhibited upregulation of *Keap1* expression and downregulation of *Nrf2* expression; furthermore, *Mn-Sod* expression levels were significantly decreased in the psoralen-treatment groups. These data suggested that the antioxidant capacity of the larvae subjected to psoralen treatment was reduced. In addition, the increased expression levels of genes encoding pro-apoptotic proteins (*p53, puma, apaf-1, caspase-9*, and *caspase-3*) and the decreased expression level of *Bcl-2* demonstrated that psoralen had induced apoptosis in zebrafish larvae through a mitochondria-dependent pathway. Moreover, the decreased expression levels of *hmgcra, pparα1*, and *fas* in this study indicated psoralen-induced abnormalities in lipid metabolism. Therefore, the embryos/larvae might have faced energy shortage during the course of development.

## Conclusion

**FIGURE 10:**
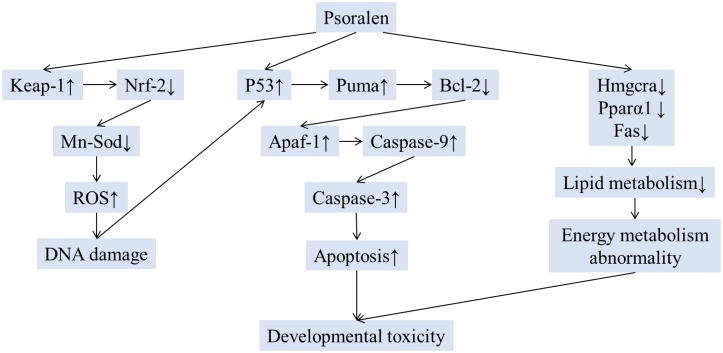
The schematic mechanism of developmental toxicity induced by psoralen.

The present study is the first report on psoralen-induced developmental toxicity in zebrafish embryos/larvae. The present results demonstrated that psoralen exerted toxic effects on the development of the heart, liver, phagocytes, and nervous system. Psoralen-treated zebrafish larvae/embryos exhibited yolk retention, pericardial edema, swim-bladder deficiency, curved body shape, inhibition of hatching, and short body length. The results also indicated that psoralen induced developmental toxicity through oxidative stress, apoptosis, and energy metabolism abnormalities (Figure 10). These conclusions should be further verified in other animal models. The detailed mechanism involved should be investigated in future studies. In this study, gene expression levels were only analyzed at 96 hpf. More detailed analysis during embryonic developmental should also be the focus of future work. Nevertheless, this study provided a better understanding of psoralen-induced developmental toxicity and the underlying molecular mechanisms.

## Ethics Statement

All experiments were carried out in compliance with standard ethical guidelines and under the control of the faculty Ethical Committee of the Biology Institute of the Shandong Academy of Sciences.

## Author Contributions

QX and KL conceived and designed the project. QX and LW analyzed the data. QX, LH, and XC wrote the manuscript. QX, YZ, HK, YS, and XW performed the experiments.

## Conflict of Interest Statement

The authors declare that the research was conducted in the absence of any commercial or financial relationships that could be construed as a potential conflict of interest.
